# Does organized sports participation in childhood and adolescence positively influence health? A review of reviews

**DOI:** 10.1016/j.pmedr.2021.101425

**Published:** 2021-05-30

**Authors:** Helga Birgit Bjørnarå, Thomas Westergren, Ellen Sejersted, Monica Klungland Torstveit, Bjørge Herman Hansen, Sveinung Berntsen, Elling Bere

**Affiliations:** a Faculty of Health and Sport Sciences, University of Agder, Kristiansand, Norway; b University Library, University of Agder, Kristiansand, Norway; c Department of Health and Inequalities & Centre for Evaluation of Public Health Measures, Norwegian Institute of Public Health, Oslo, Norway

**Keywords:** Organized sports participation, Youth sports, Children, Adolescents, Health, Systematic review of reviews

## Abstract

Sports participations have the potential for both positive and negative health outcomes. We hence aimed (i) to assess systematically reviewed associations between organized sports participation in children and adolescents and their health, and (ii) to assess qualitative syntheses of experiences among children and adolescents concerning organized sports participation and health. A search was undertaken in April 2020 across the databases MEDLINE, EMBASE, APA PsycInfo (Ovid), Scopus, SPORTDiscus (EBSCO*host*), and specialized databases for reviews. The recommended Joanna Briggs Institute approach to critical appraisal, study selection, data extraction, and data synthesis was used. Systematic reviews were included that covered children and/or adolescents aged ≤ 18 years, and adult participants with retrospective exposure to—or experiences of—organized sports participation before the age of 19 and examining health outcomes and experiences (Prospero protocol CRD 42020206677). Five reviews based mainly on cross-sectional data, two mainly on longitudinal data, and one on experimental studies were included. A causal relationship of moderate-to-high level of evidence between organized sports participation and moderate crude weight reduction accentuated by diet control and team sports was identified. Evidence of causal relationships between sports participation and reduced anxiety, and depression and increased physical activity was at a moderate level. Evidence of causal relationships between sports participation and health was of low-to-moderate level concerning obesity status (inconclusive), bone health (positive), and psychological and social health (positive and negative). Causal relationships between organized child and adolescent sports participation and health remain uncertain. Experimental and well-conducted longitudinal primary studies are highly warranted.

## Highlights

• Eight systematic reviews on organized sports participation related to health exist.
• Organized sports participation has a moderate effect on crude body weight loss.
• Dose relates to anxiety, depression, physical activity, and bone health responses.
• Associations with psychological and social health were inconclusive.
• Experimental and longitudinal population-based observational studies are needed.

## 1 Introduction

Regular participation in physical activity (PA) is associated with several physical and mental health benefits. Based on extensive research, PA recommendations have been developed, entailing at least 60 min per day of moderate-to-vigorous intensity physical activity (MVPA) for children and adolescents aged 5–17 years (World Health Organization, 2010). Complying with these recommendations is associated with increased physical fitness, reduced body fat, favorable cardiovascular and metabolic disease risk profiles, enhanced bone health, and reduced symptoms of depression and anxiety (Physical Activity Guidelines Advisory Committee, 2018, World Health Organization, 2010). Nevertheless, it has been estimated that about 80% of young persons (aged 11–17 years) globally do not meet the recommended minimum of 60 min of MVPA daily (Sallis et al., 2016).

The concept of PA incorporates a great diversity of activities including domestic, occupational, transport, and leisure-time contexts, the latter of which comprises physical exercise, sport, and unstructured recreation. Sport is commonly defined as being organized, and is usually competitive and played in a team or as an individual (Khan et al., 2012). Organized sport is one of the most popular forms of leisure-time activities worldwide, with at least one-third of children and adolescents participating in most countries (Aubert et al., 2018). Moreover, sports participation and access to sport/recreational facilities are consistently reported as correlated with PA in the literature (Sterdt et al., 2014). Participation is much higher in high-income countries, with yearly participation rates between 60% and 80% (Aubert et al., 2018), whereas there is still uncertainty concerning equal and affordable sports participation opportunities within, and between countries (Aubert et al., 2018).

The European Union (EU) White Paper on sports policy claims that EU sports policy should be evidence based (European Union, 2014). A linkage between organized sports participation and health-enhancing PA is assumed and warranted, and the EU also focuses on safeguarding children’s rights in sports, and guidelines for gender equality. To further support healthy and evidence-based sports and sports policy development, the highest level of evidence concerning the relation between participation in organized sports and health needs to be examined and reported.

Organized sports participation is associated with higher levels of PA, favorable motor development, and healthier eating habits (Nelson et al., 2011), whereas the potentially beneficial effects on weight development, bone health, cardiometabolic health, and psychosocial health are less well documented (Venetsanou et al., 2015). Furthermore, positive associations with psychological and social health factors have been reported (Clark et al., 2015, Mansfield et al., 2018), while potentially negative effects include increased consumption of alcohol and smokeless tobacco, and higher levels of stress, maltreatment, burnout, eating disorders, and overuse injuries (Bean et al., 2014, Vella, 2019). Overuse injuries and negative weight control are typically accentuated by early specialization, large amounts of practice, and a negative motivational climate (Bean et al., 2014).

Based on the abovementioned reviews, sports participation might have the potential for both positive and negative health outcomes. The evidence summarized above relies on combinations of experimental, longitudinal, and cross-sectional primary studies (Bean et al., 2014, Clark et al., 2015, Diehl et al., 2012, Kwan et al., 2014, Mansfield et al., 2018, Nelson et al., 2011, Vella, 2019, Venetsanou et al., 2015), with consequences for the strength of evidence concerning causal relationships between sports participation and health. Cultural contexts and differences, types of sports, and age as well as gender, could also possibly alter positive and negative associations between organized sports and health (Eime et al., 2013a, Eime et al., 2010, Eime et al., 2013b). Over the past two decades, the number of published systematic reviews has increased markedly, which can be confirmed by a rapid database search. Hence, the logical next step would be to systematically conduct reviews of existing systematic reviews, to provide decision makers in public health and sports policy as well as future research studies with the required evidence. Such reviews of reviews allow the findings of systematic reviews relevant to a review question to be transparently and systematically compared, and provide a clear understanding of what is known and not, as well as the certainty of such knowledge. The most characteristic feature of such a review of reviews is that the only type of evidence being considered for inclusion is at the highest level: namely, systematic reviews and *meta*-analyses (Aromataris et al., 2015).

A preliminary search in Cochrane Library, Epistemonikos, Joanna Briggs Institute (JBI) Evidence Synthesis, Database of Abstracts of Reviews of Effects (DARE), and also Prospero for any protocols, resulted in no review of reviews on the associations between organized sports participation among children and adolescents, and health. Hence, the objectives of the present systematic review of reviews were; (i) to assess the systematically reviewed relationships between organized sports participation in children and adolescents and their health; and (ii) to assess qualitative syntheses of experiences among children and adolescents concerning organized sports participation and health.

## 2 Methods

The review was guided by Aromataris et al. (2015), and the JBI’s proposed methods (Aromataris et al., 2020), and adhere to the PRISMA reporting guidelines (Moher et al., 2009). A predefined protocol has been registered in Prospero (CRD 42020206677).

### 2.1 .Inclusion criteria

#### 2.1.1 Types of participants

Reviews including children and/or adolescents aged ≤18 years, as well as adult participants with retrospective exposure to, or experiences of, organized sports participation before the age of 19 years, were considered for inclusion in the review. Reviews limited to specific populations only were not considered for inclusion.

#### 2.1.2 Exposure/phenomena of interest

We considered reviews examining organized sports participation including participation in organized general or recreational sports within sports clubs and/or extracurricular school-based sports during leisure time, for inclusion. Reviews examining school-based physical education, sport-based positive youth development programs and/or conceptualizations, or one specific sport exclusively which are beyond the scope of the current review, were not included in the review. For qualitative or mixed methods methodologies, reviews including experiences with sports participation related to health were considered for inclusion.

#### 2.1.3 Context/setting

We considered reviews irrespectively of country of origin of either the primary studies or the review for inclusion.

#### 2.1.4 Outcomes

Reviews examining health-related outcomes were considered for inclusion, including: (i) measures of weight status, anthropometrics, biomarkers, physical fitness, or other physical health indicators including reduced risk of diseases measured by objective instruments or self-reports; (ii) self-reported general, mental, and/or physical experiences and/or perceptions of the subjects’ own health; and (iii) health behaviors such as, but not limited to, physical activity and sleep, as well as food, tobacco, alcohol, or illicit drug consumption. We did not include reviews examining sports injuries, criminal behavior/records beyond illicit drug consumption, academic achievement, labor force participation, or other socioeconomic or demographic factors as the outcome in relation to organized sports participation. Reviews examining positive youth development as the outcome were also excluded, as we consider this perspective to concern a broader perspective than health-related outcomes.

#### 2.1.5 Types of studies

We included systematic reviews published in peer-reviewed scientific journals only, based on quantitative and/or qualitative systematic review study designs such as, but not limited to, *meta*-analyses, integrative reviews, metasyntheses, metaethnography, metastudies, prevalence or incidence reviews, effectiveness reviews, etiology or risk reviews, and mixed methods reviews. Expert-, narrative-, scoping-, or mapping-based or other reviews without a transparent description of search strategy, inclusion- and exclusion criteria, critical appraisal of primary studies, and a systematic analysis across studies, were not considered for inclusion as those elements are premises for a systematic review and inclusion in a review of reviews (Aromataris et al., 2015). Reviews published in English and unrestricted by the year of publication were considered for inclusion in this review of reviews.

### 2.2 Search strategy

A three-step search strategy was conducted according to the JBI reviewer manual (Aromataris et al., 2020). The initial search was done through MEDLINE (Ovid) for the following three concepts: organized sports participation, children or youth, and any health outcome (physical or mental), followed by an analysis of the text words in the title and abstract and of the index terms used to describe the retrieved papers. In terms of authors, ES executed the initial search strategy, and results were discussed with TW and HBB. The second broad search, using the identified words (text words and index terms) for the two concepts “organized youth sport leisure participation” and “systematic reviews” (quantitative or qualitative, *meta*-analysis or metasynthesis), together with the words from the predefined search filters for systematic reviews for the various databases, was then undertaken on 27–29 April 2020 by ES, across all the included databases: MEDLINE (Ovid), EMBASE (Ovid) and APA PsycInfo (Ovid), Scopus, and SPORTDiscus (EBSCO*host*). The search had no limitations regarding language or publication year. TW and HBB reviewed the search strategy. Specialized databases for reviews were also included as follows: Cochrane Library, Epistemonikos, JBI Evidence Synthesis, Database of Abstracts of Reviews of Effects (DARE), and Prospero for any protocols. Third, after screening and identifying studies eligible for inclusion, the reference lists of all included reviews were searched, and forward citation searches were performed in Scopus and Google Scholar. See Appendix 1 for the full search strategy for all databases. In addition, a hand search of the International Review of Sport and Exercise Psychology was conducted independently and blinded by TW and HBB. No additional systematic review was identified, and further hand search of targeted journals was hence considered superfluous.

### 2.3 Study screening and selection

Titles and abstracts of potentially relevant review articles were screened by TW and HBB independently and in duplicate, using the web resource Rayyan (Ouzzani et al., 2016) for organizing articles and excluding/including studies. Articles not published in English language were excluded at this stage. TW and HBB also read full-text articles for reviews considered eligible for inclusion and any discrepancies were resolved through discussion between the two reviewers.

### 2.4 Assessment of methodological quality/critical appraisal

Included reviews were assessed for methodological quality using the JBI umbrella review checklist (Aromataris et al., 2020). Checklist items were scored by TW and HBB independently and in duplicate, and checklist items three and four were also scored by ES. Studies were downgraded from ‘good’ overall quality to ‘fair’ if critical appraisal of any included primary studies was not conducted by at least two of the researchers independently. Critical appraisal of included studies is inevitable to handle study bias and flaws in the interpretation of primary study results, and poor assessment should therefore be the core element making a review of reviews interpretation of results more cautious. To assess the level of evidence for causal relationships between sports participation and health, criteria as given in Table 1 were developed through author discussions and applied henceforth.

**Table 1:** Criteria for evaluating the level of evidence in reviews examining relationships between sports participation and health.

Level of evidence for causal relationship between sports participation and health	Description of criteria
High	‘Good’ quality rating and including mostly randomized controlled studies
Moderate-to-high	‘Good’ quality rating and including mostly experimental studies
Moderate	‘Good’ quality rating and including mostly observational longitudinal studies
Low-to-moderate	‘Good’ or ‘fair’ quality rating and including mostly cross-sectional studies
Low	‘Poor’ quality rating

### 2.5 Data collection

To minimize risk of bias and maximize consistent extraction of accurate data from the included systematic reviews, the JBI data extraction tool for Systematic Reviews and Research Syntheses was used (Aromataris et al., 2020). Guided by this, HBB extracted details and data relevant to the items listed in the JBI data extraction form (see Appendix 2), where this information was available, then the extracted data were verified by TW. Any discrepancies in the data extraction and data synthesizing processes were resolved by consensus between TW and HBB.

## 3 Results

### 3.1 Study inclusion

**Fig. 1:**
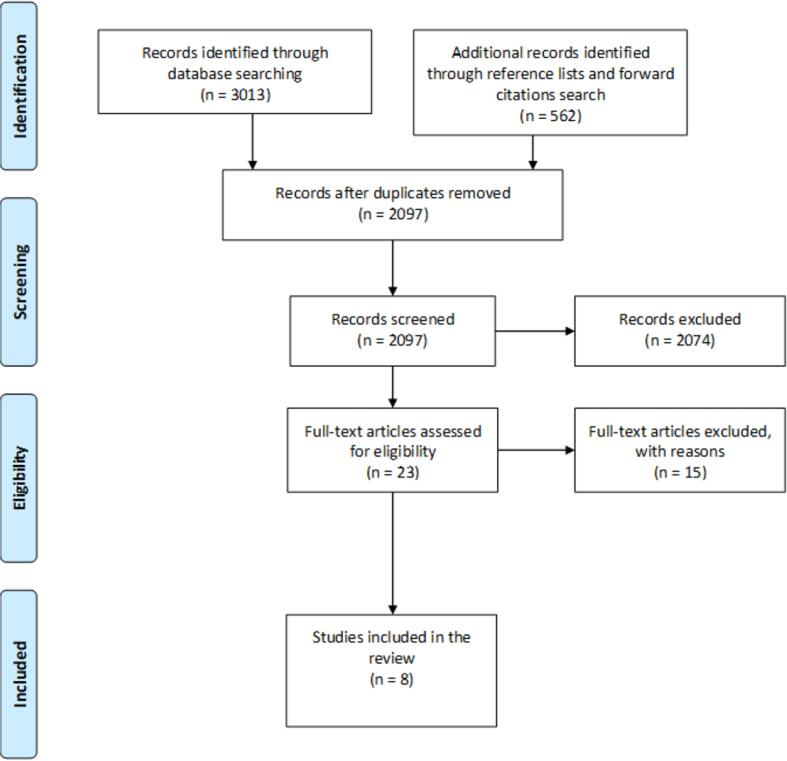
**PRISMA flow diagram**. Flow diagram of the identification, screening, eligibility, and inclusion of reviews in this review of reviews.

Database searches identified 3013 records including 1478 duplicates. Further, through reference and forward citation searches, 562 additional studies were detected for screening of titles and abstracts. Consequently, 2097 records were screened by title and abstract. After this review, 2074 articles were excluded as neither title nor abstract did match the research questions or inclusion criteria. Any study difficult to consider for inclusion based on title and abstract was assessed for full-text eligibility, meaning that 23 studies were assessed for eligibility and another 15 studies (Allender et al., 2006, Bean et al., 2014, Diehl et al., 2012, Gubbels et al., 2016, Gutierrez-Garcia et al., 2018, Holt et al., 2017, Jones et al., 2017, Kwan et al., 2014, Lisha and Sussman, 2010, Mansfield et al., 2018, Mays et al., 2011, McIntosh-Dalmedo et al., 2018, Nelson et al., 2011, Somerset and Hoare, 2018, Venetsanou et al., 2015) were excluded mainly because of poor methodological quality, meaning insufficient risk of bias assessment with a lack of critical appraisal of the included primary studies (see Appendix 3). Thus, eight studies were included in the final review (Batista et al., 2019, Eime et al., 2013b, Evans et al., 2017, Harlow et al., 2018, Lee et al., 2018, Panza et al., 2020, Tan et al., 2014). The review by Harlow et al. (2018) was included—even though it was defined as a scoping review—because it reported a search strategy, used critical appraisal, and gave outcomes in line with our inclusion criteria and systematic reviews. Fig. 1 shows a summary of the stages of study selection, according to Moher et al. (2009)

### 3.2 Methodological quality

Five studies (Batista et al., 2019, Evans et al., 2017, Harlow et al., 2018, Kim et al., 2017, Panza et al., 2020) were rated as overall good quality, and three were rated as fair (Eime et al., 2013b, Lee et al., 2018, Tan et al., 2014), as critical appraisal was not clearly conducted by two reviewers independently. Critical appraisal of the included reviews is presented in Table 2, including the overall quality ratings. All included reviews addressed primary study quality and risk of bias in relation to the review findings.

**Table 2:** Critical appraisal of the included systematic reviews.

Studies	Q1	Q2	Q3	Q4	Q5	Q6	Q7	Q8	Q9	Q10	Q11	Overall
Batista et al., 2019	Y	Y	N	Y	Y	Y	Y	Y	NA	U	Y	**Good**
Eime et al., 2013a	Y	Y	N	Y	Y	N	N	U	NA	U	Y	**Fair**
Lee et al., 2018	Y	Y	U	Y	Y	N	Y	Y	NA	Y	Y	**Fair**
Kim et al., 2017	Y	Y	U	Y	Y	Y	Y	Y	Y	Y	Y	**Good**
Harlow et al., 2018	Y	Y	Y	Y	Y	Y	Y	U	NA	U	Y	**Good**
Evans et al., 2017	Y	Y	U	Y	Y	Y	Y	Y	Y	Y	Y	**Good**
Panza et al., 2020	Y	Y	Y	Y	Y	Y	Y	Y	Y	Y	Y	**Good**
Tan et al., 2014	Y	Y	Y	Y	Y	U	Y	Y	NA	N	Y	**Fair**

List of critical assessment questions (from the JBI Critical Appraisal checklist). Q1: Is the review question clearly and explicitly stated? Q2: Were the inclusion criteria appropriate for the review question? Q3: Was the search strategy appropriate? Q4: Were the sources and resources used to search for studies adequate? Q5: Were the criteria for appraising studies appropriate? Q6: Was critical appraisal conducted by two or more reviewers independently? Q7: Were there methods to minimize errors in data extraction? Q8: Were the methods used to combine studies appropriate? Q9: Was the likelihood of publication bias assessed? Q10: Were recommendations for policy and/or practice supported by the reported data? Q11: Were the specific directives for new research appropriate? Abbreviations: Y, Yes; N, No; U, Unclear; NA, Not applicable.

### 3.3 Characteristics of included studies

Seven reviews examined organized sports participation in general (Batista et al., 2019, Eime et al., 2013b, Evans et al., 2017, Harlow et al., 2018, Lee et al., 2018, Panza et al., 2020, Tan et al., 2014) and one review examined organized sport-based interventions for weight reduction (Kim et al., 2017). Two reviews assessed obesity (Lee et al., 2018) or body weight loss (Kim et al., 2017) as outcomes, and one assessed bone strength, bone mass, and bone structure (Tan et al., 2014). We found no review including diseases, risk of diseases, or biomarkers of health for inclusion. One review assessed anxiety and depression (Panza et al., 2020) as outcomes. Three other reviews assessed other psychosocial variables as outcomes (Eime et al., 2013b, Evans et al., 2017, Harlow et al., 2018). Two reviews assessed level of PA as outcomes (Batista et al., 2019, Lee et al., 2018), but we found no studies for inclusion that assessed sedentary or screen-use time; sleep; or food, tobacco, alcohol, or illicit drug consumption.

All included reviews had large heterogeneity between studies related to study design and context, measures and analysis, and/or results. However, in one review, heterogeneity between primary study results (associations between sports participation and health) was explained by moderators (sport type and diet control; yes or no) and included in a metaregression analysis (Kim et al., 2017). Among the studies included, five were largely based on cross-sectional data (Eime et al., 2013b, Evans et al., 2017, Harlow et al., 2018, Lee et al., 2018, Tan et al., 2014), whereas two also included qualitative data (Eime et al., 2013b, Harlow et al., 2018) informing our second aim concerning experiences among children and adolescents of organized sports participation. We identified no qualitative systematic review solely addressing the second aim.

One review assessed interventional/experimental studies only (Kim et al., 2017), including 8 randomized controlled trials and 10 nonrandomized trials (Kim et al., 2017). Two reviews included a majority of longitudinal studies (Batista et al., 2019, Panza et al., 2020). In two reviews, findings were integrated by *meta*-analyses (Kim et al., 2017, Panza et al., 2020), whereas a narrative integration was conducted in four studies (Batista et al., 2019, Evans et al., 2017, Lee et al., 2018, Tan et al., 2014), and two lacked a transparent description of the analysis (Eime et al., 2013b, Harlow et al., 2018). Regarding the quality of evidence of a causal relationship between sports participation and health (see the criteria listed in Table 2), one review revealed a moderate-to-high level of evidence (Kim et al., 2017), two reviews revealed moderate levels of evidence (Batista et al., 2019, Panza et al., 2020), and five low-to-moderate levels of evidence (Eime et al., 2013b, Evans et al., 2017, Harlow et al., 2018, Lee et al., 2018, Tan et al., 2014). The characteristics of the included studies are presented in Table 3, and the full data extraction process is presented in Appendix 2.

**Table 3:** Review characteristics and findings from the systematic reviews (n = 8) included in the review of reviews on the relationships between organized participation in child and youth sport and health outcomes.

Author, year	Intervention/phenomena of interest	Participants	Outcome	Number of studies	Country of origin/context	Results/findings	Heterogeneity
Batista et al., 2019	Organized sports participation	“Children & adolescents”	Adulthood leisure PA	29 observational studies; 4 cross-sectional and 25 longitudinal	Finland, Norway, Ireland, Sweden, Belgium, Canada, Australia, and Brazil	Moderate-to-strong positive dose–response relationship (weekly frequency, practice level)	Large
Eime et al., 2013	Organized sports participation	n = 22 to > 50,000 (6–20 y)	>40 psychological and social health measures	30 studies; 21 cross-sectional and 9 longitudinal (26 quantitative, 3 qualitative, and 1 mixed method)	USA, Canada, Switzerland, Germany, UK, and Puerto Rico	Higher self-esteem, better social skills, fewer depressive symptoms, higher confidence, and higher competence	Large
Lee et al., 2018	Organized sports participation	(i) n = 21 to 71,854 (6–19 y) (ii) n = 21 to 12,188 (6–19 y)	(i) PA (ii) obesity status	(i) 27 studies; 19 cross-sectional and 8 longitudinal (ii) 17 studies; 12 cross-sectional and 5 longitudinal	(i) USA, Canada, Europe, Australia or New Zealand, and Brazil (ii) Europe, USA, and Australia	(i) Positive dose–response relationship (ii) inconclusive	Large
Kim et al., 2017	Sport-based interventions	n = 1777 (6–18 y)	Body weight loss	18 intervention studies; 8 RCTs and 10 non-RCTs	Not reported	Moderate, positive effect accentuated by sport type (team vs. individual) and diet control	Large; explained by moderators (sport type and diet control)
Harlow et al., 2018	Organized sports participation	n = 7731 boys and 7401 girls (2–6 y)	(1) psychological and emotional, (2) social, (3) cognitive or intellectual outcomes	9 studies; 2 secondary analyses of cohort data, 1 RCT, 2 experimental, 2 cross-sectional, and 2 qualitative	North America, UK, Turkey, Australia, Egypt, Greece, and Canada/Poland	Positive associations (8/9 studies), negative outcomes (2/9), some inconclusive findings	Large
Evans et al., 2017	Organized sports participation; (i) sport types, (ii) sport settings, and (iii) patterns of individual involvement	n = 27 to 13,857 (7–17 y)	Psychosocial constructs	35 studies; 19 cross-sectional, 12 longitudinal, 3 retrospective methodologies, and 1 observational	USA, Canada, England, Belgium, Sweden, Singapore, Australia, and Botswana	Inconsistent across studies, and across gender and age; dose–response relationship (negative relationship in very high involvement)	Large
Panza et al., 2020	Organized sports participation	n = 62 to 32,456 (mean age 12–18 y)	Anxiety and/or depression	29 studies; 55% with a longitudinal design and 45% cross-sectional	USA, Canada, Australia, Spain, Iceland, Japan, Nigeria, Slovenia, and one study including participants from various European countries	Small positive dose–response relationship for reduced anxiety/depression (varied across study design, age, and sex)	Large
Tan et al., 2014	Organized sports participation	n = 9 to 60 (5–18 y)	Bone strength, mass, and structure	13 observational studies (NR whether cross-sectional or longitudinal)	Not reported	Consistent positive dose–-response relationship	Large

Abbreviations: PA, physical activity; y, years; NR, not reported

### 3.4 Findings of the review

Inconclusive relationships between sports participation and *obesity status* was reported with low-to-moderate quality of evidence (Lee et al., 2018), whereas a moderate positive effect on *weight loss* based on organized sports interventions was reported with moderate-to-high quality of evidence for causal relationships (Kim et al., 2017). The effect was accentuated in team sports compared with individual sports, and in sports interventions including dietary control (Kim et al., 2017). Notably, weight was assessed as the mean of crude weight pre- and postintervention without sex, age, or height adjustments, not reporting whether included children and adolescents were underweight, of normal weight, or overweight (Kim et al., 2017). Inclusion criteria for primary studies were sports interventions including pre- and postintervention body weight data but with no criteria concerning weight status of participants pre intervention or whether body weight loss was the main intervention outcome of primary studies or not. Therefore, from the numbers presented as well as the reference list, we have assumed that the children included were underweight, of normal weight, or overweight across all studies, representing the general child population as well as e.g. children with severe obesity and children with cystic fibrosis (Kim et al., 2017). With a low-to-moderate quality of evidence for causal relationships, sports participation was reported with a positive and consistent dose–response relationship with *bone health* (Tan et al., 2014).

Organized sports participation was mainly reported with a positive association with *psychological and social variables* indicating improved health (Eime et al., 2013b, Evans et al., 2017, Harlow et al., 2018), but also with a negative association (Evans et al., 2017, Harlow et al., 2018). Negative associations were characterized as *social maladjustment* (Harlow et al., 2018) and *depression*, which increased at very high levels of involvement (Evans et al., 2017). Psychological and social constructs showing positive associations with organized sports participation included mental health, perceived health and well-being, self-concept, self-esteem, self-regulation, self-efficacy, competence, social skills, enjoyment, satisfaction, connectedness, belonging, interdependence, and group cohesion (Eime et al., 2013b, Evans et al., 2017, Harlow et al., 2018). However, in the review by Evans et al. (2017), inconclusive associations concerning enhanced developmental experiences, self-esteem, and depression, including variations across context, age, gender, and level of involvement were reported. All evidence was of low-to-moderate quality (Eime et al., 2013b, Evans et al., 2017, Harlow et al., 2018), for causal relationships, except in the review by Panza et al. (2020) who reported small positive dose–response relationships between sports participation and reduced *depression* and/or *anxiety* with a moderate quality of evidence.

Organized sports participation was reported with a strong positive dose–response relationship with coincident and subsequent *level of PA*, but with a low-to-moderate (Lee et al., 2018) and moderate (Batista et al., 2019) quality of evidence for causal relationships, respectively. The strength of evidence we apportioned reflects the observational primary studies included that were mainly cross-sectional (Lee et al., 2018) or longitudinal (Batista et al., 2019).

### 3.5 Summary of evidence

Evidence of causal relationships (Table 4) between sports participation and health were of low and low-to-moderate levels concerning obesity status, (Lee et al., 2018) bone health (Tan et al., 2014), and psychological and social health (Eime et al., 2013b, Evans et al., 2017, Harlow et al., 2018). Evidence of causal relationships between sports participation and reduced anxiety and depression (Panza et al., 2020), and increased physical activity (Batista et al., 2019) were of moderate level. The only relationship with a moderate-to-high level of evidence was that between sports participation and moderate crude weight loss among different child populations, accentuated by playing team sports with reference to individual sports, and with diet control included (Kim et al., 2017).

**Table 4:** Summary of findings of the relationship between sports participation and health in the review of reviews.

Health related to sports participation	Author(s)/year(s)/reference number	Findings	Level of evidence for causal relationship
Obesity status	Lee et al., 2018	Inconclusive	Low-to-moderate
Body weight loss	Kim et al., 2017	Moderate positive effect accentuated by team sport vs. individual & diet control	Moderate-to-high
Bone health	Tan et al., 2014	Consistent positive dose–response relationship	Low-to-moderate
Psychological and social health	Eime et al., 2013b, Harlow et al., 2018, Evans et al., 2017	Mainly positive, somewhat negative; inconclusive and variations across context, age, gender, and level of involvement	Low-to-moderate
Anxiety and depression	Panza et al., 2020	Small positive dose–response relationship	Moderate
Physical activity	Batista et al., 2019, Lee et al., 2018	Moderate to strong positive dose–response relationship	Moderate

## 4 Discussion

Five findings in this review of reviews include the following lessons learned for further evidence-informed research and sports policy. First, organized sports participation—when accompanying diet control and particularly within team sports—can be expected to reduce crude weight moderately in different populations of children and adolescents concerning weight status and/or general health. We want to emphasize that for under- or normal weight children, this might be unwanted and even harmful. There is still uncertainty concerning reductions in obesity status among children and adolescents by participation in organized sports. Second, improved bone health is associated with organized sports participation, but we cannot be sure whether organized sports improve bone health or whether children and adolescents with better bone health and physical fitness participate more commonly in such activities. Third, anxiety and depression can be expected to be slightly reduced among children and adolescents participating in organized sports, but we cannot be sure whether this is an effect of sports participation or a biased recruitment into sports leaving those with poorer mental health outside such activities. Moreover, sports participation either seems to have a potential both for positive and negative influence on psychological and social health, or at least includes both psychologically and socially healthy and unhealthy children and adolescents. Fourth, children and adolescents participating in organized sports can be expected to do more PA than their peers, but we cannot be sure whether this is an effect of sports or of biased recruitment to organized sports among more physically active children and adolescents. Fifth, we have no systematically reviewed evidence concerning additional biomarkers of health or reduced risk of diseases, or health behaviors other than PA, such as diet, sedentary time, sleeping habits, or alcohol consumption.

To establish evidence of a causal relationship, a substantial number of studies included in the original review should be based on high-quality randomized controlled trials, and heterogeneity between primary study results (if they exist) should be assessed, as conducted and reported by Kim et al. (Kim et al., 2017). Nevertheless, this field of research can be difficult to investigate through experimental studies, because organized sports participation is common, particularly in Western countries (Aubert et al., 2018). Longitudinal, larger-scale population-based observational studies might contribute to assessing relatively strong evidence supporting hypotheses of causal relationships, as suggested also within the field of PA determinants (Bauman et al., 2012). Thus, the Lancet Physical Activity Working Group has highlighted the need for longitudinal investigations of individual, interpersonal, environmental, and societal factors related to the level of PA in the general population (Bauman et al., 2012), which should also apply to organized sports participation. However, relationships assessed through cross-sectional data should be considered as hypothesis-generating associations only, and interpreted even more carefully (Bauman et al., 2012). We cannot establish evidence of causal relationships between factors from cross-sectional data. However, such data along with qualitative data might be important for further evidence-informed research and policy making. Such studies might contribute to gaining an understanding of the ‘how’ and ‘why’ sports participation relates to physical, psychological, and social health, as well as to behavioral aspects.

Relatively small reductions in weight in obese children might have positive and long-term health consequences, and help to attain a more normal weight status as they grow (Goldschmidt et al., 2013). Uncertainty concerning the relationship between obesity and organized sports participation (Lee et al., 2018), as well as uncertainty concerning weight loss per se (Kim et al., 2017), might blur recommendations for sports policy and practice concerning healthy weight control. Cautiousness should also be applied specifically concerning the risk of negative weight control associated with very high involvement in PA (Bean et al., 2014), even though such evidence has not yet been reviewed and reported systematically.

The overall pattern of associations between organized sports participation and health in this review of reviews is consistent with established knowledge on the relationship between PA and health. Engagement in PA is consistently and positively associated with physiological and social health, including more robust associations and consistency for higher intensities of participation (Poitras et al., 2016). Nevertheless, sports participation in general does not presuppose certain levels of PA, and nor does PA encompass all possible health benefits of sports participation. Organized sport is an arena for PA, as well as a social arena, and serves as an organizer concerning children’s and adolescents’ time scheduling and habitual priorities. Hence, one might also expect differences concerning associations with health for PA and for sports participation. Moreover, the negative associations between organized sports participation and psychological and social health reported here (Evans et al., 2017, Harlow et al., 2018) might not only contribute to reduced psychological and social health per se, but also reduce involvement and effort, subsequently also reduce the level of PA, and further compromise physical health. Eime et al., 2013b, Harlow et al., 2018 examined and synthesized results from primary studies to understand how sports might influence health. Both studies highlight the need for more high-quality studies to better understand the complex relationship between sports participation and health from a larger context (Eime et al., 2013b), as well as from the experiences and practices of children and adolescents themselves (Harlow et al., 2018). Such contexts and experiences might also differ between different sporting clubs and municipalities, sport types, countries, and ages as well as genders. Notably, the negative dose–response relationship between sports activities and psychological and social health in children and adolescents with the very high involvement reported by Evans et al. (2017) indicates that organized sports participation might not be considered healthy per se, but rather is an arena for health which depends on ‘who’, ‘how’, ‘how much’, and ‘where’, which is also reflected in the heterogeneity for the reviews included in this review of reviews. For instance, the accentuated effect of sports participation on weight loss by team sports and/or dietary control reported by Kim et al. (2017) highlights the fact that health may relate to the ‘how’ and ‘where’ as much as to participation per se.

This review of reviews is strengthened by a predefined protocol registered in Prospero (CRD 42020206677), and by consistent and transparent methods in concordance with JBI recommendations (Aromataris et al., 2020, Aromataris et al., 2015) and PRISMA reporting guidelines (Appendix 4). Results and subsequent recommendations are limited by the appropriate systematic reviews available and retrieved by our search strategy in the sources used, and should hence not overrule conclusions and recommendations given based on primary studies or nonsystematic reviews for relationships between organized sports participation and health not addressed here. We also recognize that the negative associations between organized sports participation and health might be linked to injuries, which were not in our scope. An additional limitation is that our results rely on the quality of original systematic review reports as well as the primary studies, where bias related to lack of clarity and transparent detailed information might have been introduced.

### 4.1 Perspective

Altogether, several systematic reviews based on a relatively large number of primary studies concerning the relationship between organized sports participation and various health outcomes have been published. However, the evidence level is generally of low-to-moderate quality and several aspects need further attention and examination. The causes behind health gains and risks remain uncertain. Sports clubs and policy makers should prioritize resources in collaboration with researchers to establish improved evidence, and organized sports participation should not be uncritically recommended to improve the health of children and adolescents.

## 5 Conclusions and recommendations

Organized sports participation has had a moderate effect on crude weight loss accentuated by dietary control and team sports, but for whom and whether weight loss was needed remains uncertain. Associations between sports participation and obesity status were inconclusive. Organized sports participation was positively associated with bone health, as well as both positively and negatively associated with psychological and social health variables. Anxiety and depression were longitudinally reduced, and level of PA increased, related to organized sports participation, but etiology was uncertain. Concerning synthesized experiences from qualitative studies, no qualitative systematic review was identified, while a few qualitative studies were included in reviews concerning positive and negative psychological and social health in relation to organized sports participation.

### 5.1 Recommendations for practice

Organized sports might be an arena for children and adolescents needing to reduce weight gain or lose weight, but there should be caution concerning the risk of negative weight control in sports. Team sports, compared with individual sports, and sports participation including a focus on appropriate diet, should be favored in recommendations from policy makers and health services for children and adolescents *needing to lose weight*.

Children and adolescents should be supported to participate in organized sports that have positive associations with physical, psychological, and social health as well as PA levels; however, health services, sports clubs, families, and individuals should also be aware of the possible psychological and social health risks within organized sports even though no evidence of causal negative health effects have been established.

### 5.2 Recommendations for research

Systematic reviews should be conducted concerning the relationships between organized sports participation in children and adolescents and biomarkers of health as well as risk of diseases. Further, primary experimental studies are clearly needed. Well-conducted longitudinal population-based observational studies starting in advance of any sports participation can also be recommended. A similar sequence for research is recommended for the area of health behaviors beyond PA, including—but not limited to—diet, sleeping, and illicit drug use and/or alcohol consumption.

Experimental studies on organized sports participation, as well as larger population- based longitudinal observational studies, including reliable data on physiological, psychological, and social health, as well as device-measured PA levels, should be conducted. Further, qualitative systematic reviews, and subsequent qualitative primary studies if needed, on the ‘how’ and ‘why’ of sports participation’s links to physical, psychological, and social health, should be conducted before designing experimental studies.

## CRediT authorship contribution statement

**Helga Birgit Bjørnarå:** Conceptualization, Data curation, Formal analysis, Investigation, Methodology, Resources, Validation, Visualization, Writing - original draft. **Thomas Westergren:** Conceptualization, Data curation, Formal analysis, Investigation, Methodology, Resources, Validation, Visualization, Writing - original draft, Writing - review & editing. **Ellen Sejersted:** Conceptualization, Data curation, Formal analysis, Investigation, Methodology, Resources, Writing - review & editing. **Monica Klungland Torstveit:** Conceptualization, Writing - review & editing, Writing - review & editing. **Bjørge Herman Hansen:** Conceptualization, Writing - review & editing. **Sveinung Berntsen:** Conceptualization, Supervision, Writing - review & editing. **Elling Bere:** Conceptualization, Data curation, Formal analysis, Investigation, Methodology, Supervision, Validation, Writing - review & editing.
