# Supplementary material for: Does organized sports participation in childhood and adolescence positively influence health? A review of reviews
Source: Prev Med Rep. 2021 May 30;23:101425. doi: 10.1016/j.pmedr.2021.101425 (PMC8190469; doi:10.1016/j.pmedr.2021.101425)
Supplement: Supplementary data 1 [file mmc1.docx]

# Search strategy umbrella review sport participation and health

## Databases

- MEDLINE (Ovid)
- EMBASE (Ovid)
- SPORTDiscus (EBSCOhost)
- APA PsycINFO (Ovid)
- Scopus

**Review - databases**

- Cochrane library (Reviews)
- JBI Evidence Synthesis (earlier name: JBI Database of Systematic Reviews and Implementation Reports)
- Epistemonikos
- The Database of Abstracts of Reviews of Effects (DARE)

## Search concepts

1. Organized sport participation
2. Children or younger person
3. Reviews

### MEDLINE (Ovid): Database: Ovid MEDLINE(R) ALL <1946 to April 24, 2020> 27.04.2020

Search Strategy:

--------------------------------------------------------------------------------

1 Youth Sports/ (366)

2 Sports/ (30033)

3 Leisure Activities/ (8565)

4 Organizations/ (11055)

5 Social Participation/ (2322)

6 recreation/ (6843)

7 3 or 4 or 5 or 6 (28314)

8 2 and 7 (1079)

9 ((sport or sports) adj7 (organized or organised or participat*)).ti,ab. (7608)

10 (organi#ed pa or (organi#ed adj3 physical activit*)).ti,ab. (269)

11 1 or 8 or 9 or 10 (8869)

12 (child* or adolescen* or teen or teens or teenager* or young* or youth* or kid or kids or boy or boys or girl or girls).ti,ab,hw. (4233465)

13 11 and 12 (5820)

14 (Review or Systematic Review or Meta-Analysis).pt. or review*.ti. or (metasynthes* or meta-synthes* or meta-analys* or systematic review* or scoping review* or Meta-Ethnography* or meta-ethnography).mp. (2928497)

15 13 and 14 (799)

### EMBASE (Ovid) Database: Embase <1974 to 2020 Week 17> 27.04.2020

Search Strategy:

--------------------------------------------------------------------------------

1 Youth Sport/ (580)

2 Sport/ (49614)

3 Leisure/ (34563)

4 Organization/ (145947)

5 Social Participation/ (5980)

6 recreation/ (18696)

7 3 or 4 or 5 or 6 (203495)

8 2 and 7 (3435)

9 ((sport or sports) adj7 (organized or organised or participat*)).ti,ab. (9435)

10 (organi#ed pa or (organi#ed adj3 physical activit*)).ti,ab. (324)

11 1 or 8 or 9 or 10 (12867)

12 (child* or adolescen* or teen or teens or teenager* or young* or youth* or kid or kids or boy or boys or girl or girls).ti,ab,hw. (4131747)

13 11 and 12 (7438)

14 Review.pt. or review*.ti. or (metasynthes* or meta-synthes* or meta-analys* or systematic review* or scoping review* or Meta-Ethnography* or meta-ethnography).mp. (3057842)

15 13 and 14 (908)

### APA PsycINFO (Ovid). Database: APA PsycInfo <1806 to April Week 3 2020> 27.04.2020

Search Strategy:

--------------------------------------------------------------------------------

1 Sports/ or Athletic Participation/ (19905)

2 Leisure Time/ or exp extracurricular activities/ or Relaxation/ (11345)

3 Organizations/ (26087)

4 Group Participation/ (2560)

5 recreation/ (5672)

6 2 or 3 or 4 or 5 (44673)

7 1 and 6 (1007)

8 ((sport or sports) adj7 (organi#ed or participat*)).ti,ab. (4329)

9 (organi#ed pa or (organi#ed adj3 physical activit*)).ti,ab. (126)

10 7 or 8 or 9 (5196)

11 (child* or adolescen* or teen or teens or teenager* or young* or youth* or kid or kids or boy or boys or girl or girls).ti,ab,hw. (1043108)

12 10 and 11 (2528)

13 limit 12 to ("0800literature review" or "0830systematic review" or 1200 meta analysis or 1300 metasynthesis) (78)

14 review*.ti. or (metasynthes* or meta-synthes* or meta-analys* or systematic review* or scoping review* or Meta-Ethnography* or meta-ethnography).mp. (187139)

15 12 and 14 (65)

16 13 or 15 (108)

17 Review-Book.dt. (118272)

18 16 not 17 (88)

### SPORTDIscus (EBSCOhost). 27.04.2020

[Link](http://search.ebscohost.com/login.aspx?direct=true&db=sph&bquery=(((TI+(sport+OR+sports)+N6+(organi%3fed+OR+participat*)+OR+TI+%26quot%3borgani%3fed+PA%26quot%3b+OR+TI+(%26quot%3borgani%3fed+N2+%26quot%3bphysical+activit*))+OR+(AB+(sport+OR+sports)+N6+(organi%3fed+OR+participat*)+OR+AB+%26quot%3borgani%3fed+PA%26quot%3b+OR+AB+(%26quot%3borgani%3fed+N2+%26quot%3bphysical+activit*))+OR+(SU+%26quot%3bSPORTS+participation%26quot%3b))+AND+(child*+OR+adolescen*+OR+teen+OR+teens+OR+teenager*+OR+young*+OR+youth*+OR+kid+OR+kids+OR+boy+OR+boys+OR+girl+OR+girls))+AND+((TI+review*)+OR+(metasynthes*+OR+%26quot%3bmeta-synthes*%26quot%3b+OR+%26quot%3bmeta-analys*%26quot%3b+OR+%26quot%3bsystematic+review*%26quot%3b+OR+%26quot%3bscoping+review*%26quot%3b+OR+%26quot%3bMeta-Ethnography*%26quot%3b+OR+%26quot%3bmeta-ethnography%26quot%3b))&type=1&searchMode=Standard&site=ehost-live)

| **#** | **Query** | **Results** |
| --- | --- | --- |
| S1 | TI (sport OR sports) N6 (organi?ed OR participat*) OR TI "organi?ed PA" OR TI ("organi?ed N2 "physical activit*") | 3,548 |
| S2 | AB (sport OR sports) N6 (organi?ed OR participat*) OR AB "organi?ed PA" OR AB ("organi?ed N2 "physical activit*") | 11,602 |
| S3 | SU "SPORTS participation" | 4,524 |
| S4 | S1 OR S2 OR S3 | 16,714 |
| S5 | child* or adolescen* or teen or teens or teenager* or young* or youth* or kid or kids or boy or boys or girl or girls | 247,748 |
| S6 | S4 AND S5 | 5,809 |
| **S7^[[1]](#footnote-1)^** | **TI review*** | **31,054** |
| S8 | metasynthes* or "meta-synthes*" or "meta-analys*" or "systematic review*" or "scoping review*" or "Meta-Ethnography*" or "meta-ethnography" | 14,581 |
| S9 | S7 OR S8 | 37,239 |
| S10 | S6 AND S9 | 152 |

### Scopus 27.04.2020^[[2]](#footnote-2)^

**Advanced search:**

( ( TITLE-ABS-KEY ( ( sport OR sports ) W/6 ( organi?ed OR participat* ) ) ) OR ( TITLE-ABS-KEY ( "organi?ed PA" ) ) OR ( TITLE-ABS-KEY ( organi?ed W/2 "physical activit*" ) ) ) AND ( TITLE-ABS-KEY ( child* OR adolescen* OR teen OR teens OR teenager* OR young* OR youth* OR kid OR kids OR boy OR boys OR girl OR girls ) ) AND ( DOCTYPE ( re ) OR TITLE ( review* ) OR TITLE-ABS-KEY ( metasynthes* OR "meta-synthes*" OR "meta-analys*" OR "systematic review*" OR "scoping review*" OR "Meta-Ethnography*" OR "meta-ethnography" ) )

**882 hits**

## Additional database search 29.04.2020

### Cochrane library

Search Name: organized-sport-participation-child-younger-health

Date Run: 29/04/2020 13:23:19

Comment:

<https://www.cochranelibrary.com/web/cochrane/advanced-search/search-manager?search=3666503>

ID Search Hits

#1 MeSH descriptor: [Youth Sports] this term only 9

#2 MeSH descriptor: [Sports] this term only 858

#3 MeSH descriptor: [Leisure Activities] this term only 254

#4 MeSH descriptor: [Organizations] this term only 16

#5 MeSH descriptor: [Social Participation] this term only 98

#6 MeSH descriptor: [Social Participation] this term only 98

#7 #3 OR #4 OR #5 OR #6 365

#8 #2 AND #7 12

#9 ((sport or sports) NEAR/6 (organi?ed or participat*)):ti,ab 465

#10 (organi?ed pa or (organi?ed NEAR/2 (physical NEXT activit*))):ti,ab 74

#11 #1 OR #8 OR #9 OR #10 545

#12 ((child* or adolescen* or teen or teens or teenager* or young* or youth* or kid or kids or boy or boys or girl or girls)):ti,ab,kw 295941

#13 #11 AND #12 314 **(6 – reviews)**

### JBI Evidence Synthesis (earlier name: JBI Database of Systematic Reviews and Implementation Reports)

Not so sophisticated search functionalities

The systematic reviews from JBI is indexed in both MEDLINE and CINAHL databases

Simple phrase search:

organized sport" 1 hit

<https://journals.lww.com/jbisrir/pages/results.aspx?fs=%5b%7b%22Operator%22%3a%22and%22%2c%22Field%22%3a%22All+Fields%22%2c%22Key%22%3a%22%5c%22organized+sport%5c%22%22%7d%5d>

"organized sports" 3 hits

<https://journals.lww.com/jbisrir/pages/results.aspx?fs=%5b%7b%22Operator%22%3a%22and%22%2c%22Field%22%3a%22All+Fields%22%2c%22Key%22%3a%22%5c%22organized+sports%5c%22%22%7d%5d>

**Total: 4 hits**

### Epistemonikos 29.04.2020

Do not support proximity operator, will broaden the search

Simple search form:

(organized OR organised) AND (sport OR sports OR "physical activity" OR PA) AND (child* or adolescen* or teen or teens or teenager* or young* or youth* or kid or kids or boy or boys or girl or girls)

[Link](https://www.epistemonikos.org/en/search?&q=(organized%20OR%20organised)%20AND%20(sport%20OR%20sports%20OR%20%22physical%20activity%22%20OR%20PA)%20AND%20(child*%20or%20adolescen*%20or%20teen%20or%20teens%20or%20teenager*%20or%20young*%20or%20youth*%20or%20kid%20or%20kids%20or%20boy%20or%20boys%20or%20girl%20or%20girls))

Limit:

- Broad synthesesis: 6
- Systematic reviews: 86
- **Total: 92**

**Prospero – protocol**

organized AND (sport OR sports OR physical activity OR PA) AND (child* or adolescen* or teen or teens or teenager* or young* or youth* or kid or kids or boy or boys or girl or girls)

**82 hits**

### The Database of Abstracts of Reviews of Effects (DARE)

Results for: (organized ) AND (Sport OR sports OR physical activity OR PA) AND (child* or adolescen* or teen or teens or teenager* or young* or youth* or kid or kids or boy or boys or girl or girls) IN DARE: 0 hits

## **Result hits:**

| **Database search 27-29.04.2020** | **Number** |
| --- | --- |
|  |  |
| MEDLINE (Ovid) | 799 |
| EMBASE (ovid) | 908 |
| PsycINFO (ovid) | 88 |
| SPORTDiscus (Ovid) | 152 |
| Scopus | 882 |
| cochrane | 6 |
| jbi | 4 |
| epistemonikos | 92 |
| prospero | 82 |
| **Total** | **3013** |
| Duplicates | 1478 |
| **Uniqe, Rayyan screening TI-AB** | **1535** |

1. Possible to narrow – missing the narrative reviews: Vella, S. A. (2019). Mental Health and Organized Youth Sport. Kinesiology Review, 8(3), 229-236. Weiss MR. Youth Sport Motivation and Participation: Paradigms, Perspectives, and Practicalities. Kinesiology Review. 2019;8(3):162-170 -If searching with OR AB review- extra hits 320 - [link](http://search.ebscohost.com/login.aspx?direct=true&db=sph&bquery=((((TI+(sport+OR+sports)+N6+(organi%3fed+OR+participat*)+OR+TI+%26quot%3borgani%3fed+PA%26quot%3b+OR+TI+(%26quot%3borgani%3fed+N2+%26quot%3bphysical+activit*))+OR+(AB+(sport+OR+sports)+N6+(organi%3fed+OR+participat*)+OR+AB+%26quot%3borgani%3fed+PA%26quot%3b+OR+AB+(%26quot%3borgani%3fed+N2+%26quot%3bphysical+activit*))+OR+(SU+%26quot%3bSPORTS+participation%26quot%3b))+AND+(child*+OR+adolescen*+OR+teen+OR+teens+OR+teenager*+OR+young*+OR+youth*+OR+kid+OR+kids+OR+boy+OR+boys+OR+girl+OR+girls))+AND+((AB+review*+OR+TI+review*)+OR+(metasynthes*+OR+%26quot%3bmeta-synthes*%26quot%3b+OR+%26quot%3bmeta-analys*%26quot%3b+OR+%26quot%3bsystematic+review*%26quot%3b+OR+%26quot%3bscoping+review*%26quot%3b+OR+%26quot%3bMeta-Ethnography*%26quot%3b+OR+%26quot%3bmeta-ethnography%26quot%3b)))+NOT+(TI+review*+OR+metasynthes*+OR+%26quot%3bmeta-synthes*%26quot%3b+OR+%26quot%3bmeta-analys*%26quot%3b+OR+%26quot%3bsystematic+review*%26quot%3b+OR+%26quot%3bscoping+review*%26quot%3b+OR+%26quot%3bMeta-Ethnography*%26quot%3b+OR+%26quot%3bmeta-ethnography%26quot%3b)&type=1&searchMode=Standard&site=ehost-live) [↑](#footnote-ref-1)
2. List of document types Scopus: <https://service.elsevier.com/app/answers/detail/a_id/11236/supporthub/scopus/> [↑](#footnote-ref-2)
