# Supplementary material for: Does organized sports participation in childhood and adolescence positively influence health? A review of reviews
Source: Prev Med Rep. 2021 May 30;23:101425. doi: 10.1016/j.pmedr.2021.101425 (PMC8190469; doi:10.1016/j.pmedr.2021.101425)
Supplement: Supplementary data 2 [file mmc2.docx]

| **Data extraction form, umbrella reviews** |
| --- |

| Study details | 1 |
| --- | --- |
| Title | **Participation in sports in childhood and adolescence and physical activity in adulthood: A systematic review.** |
| Author/year | **Batista et al., 2019.** |
| Objectives | To analyze the association between participation in sports (PS) in childhood and adolescence and levels of PA in adulthood. |
| Type of review | Systematic review. |
| Participant details (characteristics, total number) | 29 primary studies included, number of participants not reported. |
| Setting and context | Not explicitly reported – only reported that initial eligible criteria were studies including healthy samples, and being published in English, Spanish or Portuguese. |
| Description of intervention/phenomena of interest | PS in childhood and adolescence, operationalized as specific aspects of the PS, such as: “frequency”, established based on days or sessions per week; “persistence in PS”, generally accounted for in years; “type and number of sports practiced”, classified as individual or collective/team; and “PS level reached”, identified as recreational, competitive (club, regional, national), or elite/professional. |
| Search details |  |
| Number of databases sourced and searched | Six databases: Medline (PUBMED), Web of Science, Scopus, and SPORTDiscus, in addition to the Latin American databases LILACS and SciELO. |
| Date range of database searching | The entire period of existence of the databases until December 2018. |
| Publication date range of included studies | 1996 to 2018. |
| Number and types of studies | 29 observational studies; 4 cross-sectional and 25 longitudinal. |
| Country of origin of included studies | Finland, Norway, Ireland, Sweden, Belgium, Canada, Australia, and Brazil. |
| Appraisal |  |
| Instruments used to appraise the primary studies and their quality | Adapted STROBE; including 15 items. |
| Appraisal rating | Each item met accounted for 1 point; further categorized into: 0-5 points = low quality; 6-10 points = moderate quality; and 11 to 15 points = high quality. Studies that achieved high quality were included. |
| Analysis |  |
| Methods of analyses | Not explicitly reported, only stating that ‘the strength of the association was indicated according to the interpretation of the results and discussion by the authors of the studies (weak, moderate, strong), not establishing numerical cut-offs due to the great variability of statistical analyses used’. |
| Outcome(s) assessed | Adulthood leisure PA; habitual PA; MVPA; leisure MVPA; VPA (times and hour/week); MVPA (hour/week). |
| Results/findings |  |
| Significance/direction | No relationship between PS in childhood and adolescence and PA in adulthood was found in three (10%) studies, and no inverse relationships were identified. A weak, positive relation was found in two (7%) studies, a moderate relation in 18 (62%) studies, and a strong relation in six (21%) studies. Also, it was found that higher weekly frequencies of PS, at least three years of PS, and the highest level of practice and/or competition reached were related to higher levels of PA in adulthood. The authors concluded that there is a positive association considered moderate to strong between PS in childhood and adolescence and PA in adulthood. |
| Heterogeneity | Authors reported great variability of statistical analyses used in the primary studies. |
| *Comments:* |  |

| Study details | 2 |
| --- | --- |
| Title | **A systematic review of the psychological and social benefits of participation in sport for children and adolescents: informing development of a conceptual model of health through sport.** |
| Author/year | **Eime et al., 2013.** |
| Objectives | Worded differently, yet clear; “Firstly, this paper presents the results of a systematic review investigating the psychological and social benefits of participation in sport for children and adolescents. Secondly, the information obtained in the systematic review has been used to develop the conceptual model of Health through Sport, for children and adolescents.” |
| Type of review | Systematic review. |
| Participant details (characteristics, total number) | School age children, range 6-20 years (great variations in age across studies, generally 18 years or less); samples ranged from 22 to over 50,000 participants. |
| Setting and context | Community; several contexts of participation in sports: extracurricular activities; team sport; school or club sport; and sports in general. |
| Description of intervention/phenomena of interest | Sports participation in children and adolescents (<18y). |
| Search details |  |
| Number of databases sourced and searched | 14 databases: AU SPORT, AusportMed, CINAHL, Cochrane Library, EBSCHOHost Research Databases, Health Collection, Informit, Medline Fulltext, PsycARTICLES, Psychology and Behavioral Sciences Collection, PsycINFO, PubMed, Scopus, SPORTDiscus Fulltext |
| Date range of database searching | January 1990 to May 2012. |
| Publication date range of included studies | 1993-2011. |
| Number and types of studies | 30 included studies; 26 quantitative, 3 qualitative and one mixed method, of which 0 RCTs, 21 cross-sectional and 9 longitudinal study designs. |
| Country of origin of included studies | US:21 studies; Canada:4; Switzerland:3, Germany, UK and Puerto Rica:1. |
| Appraisal |  |
| Instruments used to appraise the primary studies and their quality | The Downs and Black checklist; 27 items, yet not all items were applicable due to the observational design of the 26 quantitative studies. |
| Appraisal rating | A summary quality score was calculated for each paper (except the two qualitative papers for which only five items were applicable) by expressing the number of compliant items as a percentage of the number of applicable  items. Scores ranged from 33% to 88%, and insights from the scoring process was used for discussing study quality. |
| Analysis |  |
| Methods of analyses | Not explicitly reported, yet studies were combined narratively. |
| Outcome(s) assessed | In total 40 different psychological and social health measures were reported, most commonly related to: (i) psychosocial functioning and emotional well-being (n=6 studies), (ii) risk of depression and mental ill health (n=5), (iii) developmental aspects/behavior (n=4), (iv) social anxiety and shyness (n=3), (v) self-esteem (n=3) and (vi) suicidal behavior (n=3). |
| Results/findings |  |
| Significance/direction | The most common ***positive outcomes*** were higher self-esteem (n=6), better social skills (n=5), fewer depressive symptoms (n=4), higher confidence (n=3) and higher competence (n=3 studies) among sport participants compared with non-sport participants. Sport may be associated with improved psychosocial health above and beyond improvements attributable to participation in PA. Specifically, team sport seems to be associated with improved health outcomes compared to individual activities, due to the social nature of the participation. |
| Heterogeneity | Included studies entailed a wide range of aims, focuses, measurement tools and indicators of both sport participation and health outcomes, which limited the extent of synthesis and precluded meta-analysis. |
| *Comments:* |  |

| Study details | 3 |
| --- | --- |
| Title | **The role of youth sports in promoting children's physical activity and preventing pediatric obesity: a systematic review.** |
| Author/year | **Lee et al., 2018.** |
| Objectives | To systematically review the impact of youth sports participation on (i) daily PA participation and (ii) pediatric obesity in children aged 6 to 19 years. |
| Type of review | Systematic review. |
| Participant details (characteristics, total number) | (i)sample sizes ranged from 21 to 71,854, subjects aged 6 to 19 years. More than half of the studies (n= 16) targeted adolescents only (13 to 19 y), while five studies were on children only, and three studies included both adolescents and children.  (ii) Sample sizes ranged from 21 to 12,188; ages 6 to 19 years. seven targeted adolescents only, eight exclusively children, and two studies of both populations. |
| Setting and context | (i)Seven studies took place in school-based sport settings, four studies in community-based settings, six studies  used both, and 10 studies did not report specific sports settings.  (ii) Not reported. |
| Description of intervention/phenomena of interest | Participation in youth sports; operationalized as both organized and nonorganized competitive sports that are practiced among children and adolescents out of physical education classes. Additionally, “youth sports” included any activities or PA interventions that employed sports. Thus, extracurricular community PA interventions such as soccer teams, after school sports (both at school and outside school), and boy scouts were included as youth sports. |
| Search details |  |
| Number of databases sourced and searched | Six databases: Web of Science, Academic Search Premier, Google Scholar, Pub Med, PsycInfo, and ERIC. |
| Date range of database searching | Up to January 2014, from when is not reported. |
| Publication date range of included studies | Not reported. |
| Number and types of studies | (i)27 studies investigated the influence of youth sport on children’s PA; three studies had an adult sample adopting a retrospective design. Eight studies implemented a longitudinal design (including one intervention design), while 19 were cross-sectional.  (ii)17 studies examined the association between youth sport participation and obesity status;12 studies were cross- sectional, five were longitudinal. |
| Country of origin of included studies | (i)17 were from the United States and Canada, seven were from Europe, two were from Australia or New Zealand, and one from Brazil.  (ii) Nine studies were conducted in Europe, seven in the US, and one in Australia. |
| Appraisal |  |
| Instruments used to appraise the primary studies and their quality | The quality of study findings and methodology was assessed “based on 9 items adapted from prior work”, and considered sample randomization, comparison group, dose of sport participation, pre-post assessment, retention, power analysis, use of valid measures, follow-up, and handling of missing data. |
| Appraisal rating | Scoring methodology not reported, only referring to prior work. |
| Analysis |  |
| Methods of analyses | Not explicitly reported, yet studies are combined narratively. |
| Outcome(s) assessed | (i)PA-related outcomes (e.g., adherence to PA, status of meeting the recommended PA level, leisure time PA, total daily energy expenditure, and/or time spent in MVPA)  (ii)obesity-related outcomes (e.g., BMI, body fat percentage, skinfold, and/or waist circumference); cross-sectional effect or longitudinal effect. |
| Results/findings |  |
| Significance/direction | (i)15 studies indicated that greater amounts and frequency of PS associated with a more physically active lifestyle in youth and/or later in adulthood, with ORs (n=7) ranging from 1.74 [CI 95% 1.13, 2.67] to 13.2 [CI 95% 9.4, 18.7]. These seven studies investigated the following outcomes: MVPA frequency, meeting PA guidelines or not, frequencies of walking and cycling, or future MVPA levels. One study indicated no difference over time in MVPA between a sports-program intervention group and control group. One study found that individuals enrolled in a sports club at a younger age were more likely to be physically active than those who joined at an older age (r-0.18, p <0.01). It was also suggested that youth involved in sports demonstrated greater daily energy expenditure. The findings concerning whether community-based sport and school sport had a different effect on PA were inconclusive, yet some studies reported participation in community sports to be a stronger predictor of MVPA.  (ii) Results for the association between youth sport participation and obesity were mixed. Several studies reported an inverse association with pediatric obesity (fewer negative obesity-related outcomes), yet six studies revealed no significant relations, or only partial or weak relations in subgroup comparisons. Hence, one cannot conclude that significant relations between youth sport participation and pediatric obesity exists, and most of studies employed a cross-sectional design. |
| Heterogeneity | Large heterogeneity in study setting and design. |
| *Comments:* |  |

| Study details | 4 |
| --- | --- |
| Title | **Sport-based physical activity intervention on body weight in children and adolescents: a meta-analysis.** |
| Author/year | **Kim et al., 2017.** |
| Objectives | (i)To evaluate the overall impact of sport-based interventions on body weight among children and adolescents.  (ii)To determine the influence of moderators (gender (boy, girl or both), age (children or adolescents), intervention length (less than 3 months, 3–6 months or more than 6 months), sport type (individual or team) and diet control (yes or no)) on the analyzed studies. |
| Type of review | Meta-analysis. |
| Participant details (characteristics, total number) | In total 1777 participants, aged 6-18 years. |
| Setting and context | Not reported. |
| Description of intervention/phenomena of interest | Sport-based interventions. |
| Search details |  |
| Number of databases sourced and searched | Seven databases: Academic Search Complete, Education Source, ERIC, Medline, ProQuest Database, PsycINFO and SportDiscus |
| Date range of database searching | From January 2000 to April 2015. |
| Publication date range of included studies | Year 2000-2014. |
| Number and types of studies | 18 intervention studies; eight classified as RCTs, 10 as none RCTs. |
| Country of origin of included studies | Not reported. |
| Appraisal |  |
| Instruments used to appraise the primary studies and their quality | The Downs and Black checklist; 27 items across four sections including reporting, external validity, internal validity, and power providing an overall score for study quality. The total maximum score was 28 (1 point per each question except question 5, giving 2 points). |
| Appraisal rating | Higher score indicated higher methodological quality (maximum score 28). Any study with low methodological quality (i.e., Z-score less than −2.58 or greater than 2.58) was further scrutinized to determine exclusion from the study or not. The methodological quality of the included studies was robust (mean ± standard deviation (SD) 22.11 ± 2.40) ranging from 16 to 27. No studies had quality scores outside three standard deviations of the mean. Average scores for each measurement domain were: (a) reporting (9.22 of 11), (b) external validity (1.72 of 3), (c) internal validity (10.17 of 13) and (d) power: (1 of 1). |
| Analysis |  |
| Methods of analyses | A random effects model was used to calculate the mean ES and 95% CI. Based on Cohen’s definition, ESs were classified as small = 0.2, medium = 0.5 and large = 0.8. Cochran’s Q statistics was used to examine the heterogeneity of the mean ES. The moderator analyses were conducted to examine the effects of each moderator on reducing weight. The publication bias was analyzed via visual inspection of a funnel plot and Egger’s test of the regression intercept. |
| Outcome(s) assessed | Body weight. |
| Results/findings |  |
| Significance/direction | ES calculations showed that the treatment effect was statistically significant (Cohen’s d (ES) = 0.52, 95% CI = 0.08, 0.95, P = 0.021) using a random effects model. Based on Cohen’s criteria, the weighted mean ES was moderate in magnitude. The Q statistic for the sport type (individual or team) and diet control (yes or no) was statistically significant. Based on the funnel plot, studies were symmetrically distributed indicating that the potential for publication bias was marginal across the studies. Egger’s test of regression intercept was −1.77 (two-tailed P-value = 0.432), hence there was no evidence of publication bias. The results indicated that the use of sport as an intervention strategy has a moderate, positive effect on body weight loss. The cumulative evidence suggested that only sport type (team sport vs. individual) and diet control significantly influence body weight loss among the moderators (i.e. not age, gender of participants, intervention length). |
| Heterogeneity | Some heterogeneity in ESs, explained by the moderator sports type (team vs individual) and diet control (yes vs no). |
| *Comments:* |  |
|  |  |
| Study details | **5** |
| Title | **Should toddlers and preschoolers participate in organized sport? A scoping review of developmental outcomes associated with young children’s sport participation.** |
| Author/year | **Harlow et al., 2018.** |
| Objectives | To investigate and synthesize scholarly articles examining psychological, emotional, social, cognitive, or intellectual developmental outcomes of organized sport involvement of children between 2 and 5 years of age. |
| Type of review | Scoping review. |
| Participant details (characteristics, total number) | In total, 7731 males and 7401 females were included in the nine studies, aged 3-6 years and primarily Caucasian, followed by Hispanic. |
| Setting and context | Preschoolers participating in dance (i.e. general, ballet, folk), team sports, or individual sports (including t-ball, swimming, hydro-gymnastics, judo, tennis, karate, soccer, basketball, and track and field), while two studies did not specify the type of sports. |
| Description of intervention/phenomena of interest | Participation in organized sport programs (defined as PAs that take place outside of school hours, and are led  by a coach or instructor, entailing rules, a degree of effort and strategy, and being practiced regularly). |
| Search details |  |
| Number of databases sourced and searched | Nine electronic bibliographic databases and citation indexes were searched; PsycINFO, Sociological Abstracts, Educational Abstracts, SPORT Discus, Scopus, Web of Science, ERIC, Cochrane, and MEDLINE (Ovid). |
| Date range of database searching | 1996-June 2017. |
| Publication date range of included studies | 1996-2016. |
| Number and types of studies | Nine studies, of which seven utilized quantitative methodologies, with two performing secondary analyses of cohort data, one entailed a randomized experimental design, two used non-randomized experimental designs, and two were cross-sectional analyses. |
| Country of origin of included studies | Three of the studies drew from samples which originated in North America, one study from each of the UK, Turkey, Australia, Egypt, and Greece, while one study had authors from both Canada and Poland, but did not specify where data was collected. |
| Appraisal |  |
| Instruments used to appraise the primary studies and their quality | Version 2011 of the Mixed Methods Appraisal Tool (MMAT), being deemed appropriate to appraise and describe the methodological quality of qualitative, quantitative, and mixed-methods studies. |
| Appraisal rating | Overall, two studies met 100% of the MMAT methodological quality criteria, one study met 75%, four met 50%, and two met 25% of the criteria. None were excluded due to the small number of studies included in the review and the exploratory nature of the study (scoping review). |
| Analysis |  |
| Methods of analyses | Not explicitly reported, and methods used to combine studies is unclear. It is only stated that, concerning the primary studies (seven quantitative studies), methods of data analysis varied including MANOVA, T-Test, linear  regression, 2-way ANOVA, and least-squared regression. One study used qualitative methodology, yet the method of data analysis was not reported or discernable in the text, and one study was a mixed-methods study. |
| Outcome(s) assessed | Study outcomes were grouped into three main developmental categories; (1) psychological and emotional, (2) social, and (3) cognitive or intellectual, with sub-categorization within each of these broader domains. Some studies  addressed more than one outcome, hence they were included in more than one category. |
| Results/findings |  |
| Significance/direction | Overall, eight studies (out of nine) reported positive associations between preschooler sport participation and developmental outcomes. Specifically, positive associations were found with development of social skills such as social competence and social adjustment, and psychological outcomes related to increased positive behaviors (e.g. responsibility, courage, respect, self- discipline, independence, etc.), and fewer emotional problems (e.g. feeling worried or anxious, withdrawn or depressed), conduct issues (e.g. obedience or fighting, interpersonal issues), or hyper-inattention problems (e.g. being restless or overactive). Psychological outcomes also included the development of perceived competence, which included recognition of abilities in relation to others, and self-regulation. It was also suggested that sport participation could enhance cognitive-intellectual developmental outcomes related to mathematical and linguistic skills. However, negative outcomes were outlined in two studies, and highlighted some inconclusive findings, e.g. one study suggested that sport participation contributed to social maladjustment, being reflected by quarrelling with friends, life dissatisfaction, complaining, giving up, lack of motivation, and being disruptive during sport practice. The authors conclude that research with stronger quality, rigor, and methodological design is needed before conclusive findings or generalizations can be inferred. |
| Heterogeneity | There was significant heterogeneity in study design and methods as well as frequency of sport participation (from zero times per week to almost every day), which limited the ability to appraise quality and synthesize findings uniformly across studies. |
| *Comments:* |  |

| Study details | 6 |
| --- | --- |
| Title | **Are all sport activities equal? A systematic review of how youth psychosocial experiences vary across differing sport activities.** |
| Author/year | **Evans et al., 2017.** |
| Objectives | (i)To systematically search available literature and explore how the design of sport activities relates to psychosocial outcomes.  (ii)To form a conceptual depiction of the ways that youth sport activities are studied to provide a roadmap for  researchers and practitioners to study and adapt sport activities to promote ideal psychosocial outcomes. |
| Type of review | Systematic review. |
| Participant details (characteristics, total number) | Samples ranged from 27 to 13 857 (Median=312) participants, aged 7-17 years (90% of included samples), all <20 years. |
| Setting and context | Participants completed surveys in school settings (eg, large school-based studies), or within sport settings as members of sport groups. |
| Description of intervention/phenomena of interest | Different forms/design of youth sport activities, according to: (i)sport types, (ii)sport settings and (iii)patterns of individual involvement. |
| Search details | A systematic search protocol was designed a priori, and followed the PRISMA-guidelines. |
| Number of databases sourced and searched | Five databases were searched; SPORTDiscus, CINAHL and ERIC via EBSCOhost; MEDLINE via PubMed and PsycINFO via SCOPUS + Google Scholar as a supplemental step. Search strategies were designed uniquely for each database, yet the search queries combined four groups of terms. |
| Date range of database searching | January 1980 and May 2016. |
| Publication date range of included studies | Not explicitly reported. |
| Number and types of studies | 35 studies were included, having the following designs; cross-sectional designs (n=19), longitudinal designs (including two to four time points; n=12), retrospective methodologies to examine sport histories (n=3) and an observational study of athlete interactions (n=1). |
| Country of origin of included studies | Research was conducted across North America (ie, USA, Canada), Europe (eg, England, Belgium, Sweden), the South  Pacific region (ie, Singapore, Australia) and Africa (ie, Botswana). |
| Appraisal |  |
| Instruments used to appraise the primary studies and their quality | An adapted coding tool, based on previous research, was developed and used by two of the authors as a template for initially recording the information from each article, including: citation details, country of participants, guiding theory, study design, psychosocial constructs studied, sport activity(ies) and related operationalization, sample characteristics, study findings and analyses. This coding tool was also used to assess risk of bias in relation to study methods, analyses, and reporting. As an example of the adaptation, the Downs and Black tool used by Eime et al (2013) was adapted by removing items that were only relevant for interventions, involving those regarding intervention descriptions, adverse events, and blinding participants. In addition to the nine items retained from the Downs and Black tool (ie, items 1, 3, 6, 7, 10, 16, 18, 25, 27), four items regarding how studies operationalized sport activities were added to the methodological items. The resulting coding sheet included 13 items that were each coded using a binary 1/0 rating, with items primarily covering methodology and analysis. |
| Appraisal rating | Greater values on the 13-item scale represented lower risk for bias, i.e. higher quality. Assessments of risk of bias in methods and analysis ranged from 7 to 12, with most studies (n=25) fulfilling 9 or more of the risk of bias items. |
| Analysis |  |
| Methods of analyses | The first step within the synthesis process was to organize studies into groups based on the approach used to study sport  activities. The second step was to review studies from each resulting group to summarize evidence. This involved a configurative approach by describing the nature of research and approaches to operationalize activities, along with an aggregative approach to identify the strength of evidence and account for consistency of findings across studies. |
| Outcome(s) assessed | Psychosocial constructs included measurement tools representing *psychosocial health and well-being* (eg, perceived well-being, quality of life, mental health, depression, anxiety, suicidal ideation), *sport-specific or general self-concept* (eg, self-esteem, self-concept, body dissatisfaction, perceived competence), *moral beliefs* (eg, moral reasoning, moral decision-making, sports-personship), *development of positive assets* (eg, developmental experiences, psychological skills), *sport-specific motives and intentions* (eg, behavioral regulation, sport enjoyment, intentions to return, psychological need satisfaction, achievement goal orientation) and *sport group environments* (eg, perceived interdependence, group cohesion). |
| Results/findings | A conceptual model was generated, consisting of three ways of studying sport activities; (i)sport types, (ii)sport settings, and (iii)patterns of individual involvement. |
| Significance/direction | Current body of evidence indicates that psychosocial outcomes of sport involvement may be optimized when youth engage in certain forms of sport activities. That is, youth involved in sport groups featuring greater interdependence reported outcomes such as enhanced developmental experiences and self-esteem, as well as lower depression and poorer moral reasoning. These effects were not consistent across all studies and varied across gender and age. Further, youth who were more deeply involved in sport overall (i.e. increased number of sport teams or higher weekly involvement) reported outcomes such as lower depression and higher self-esteem, although these effects varied across studies, and there was evidence that those with very high involvement (e.g. >17 hours of sport involvement per week) experienced higher depression. |
| Heterogeneity | There was large heterogeneity, including varying definitions of sport activities and diverse outcomes, resulting in complex combinations of findings. |
| *Comments:* | According to the authors, most effects uncovered were dependent on moderating factors (age or gender), were inconsistent across studies or had only been examined in a limited number of studies. |
|  |  |
| Study details | **7** |
| Title | **Adolescent Sport Participation and Symptoms of Anxiety and Depression: A Systematic Review and Meta-Analysis.** |
| Author/year | **Panza et al., 2020.** |
| Objectives | To explore the relationship between adolescent organized sport participation and self-reported symptoms of anxiety and depression. |
| Type of review | Systematic review and meta-analysis. |
| Participant details (characteristics, total number) | In total 122,056 participants were included, samples ranging from 62 to 32,456 participants, with a mean age between 12 and 18 years and with all participants older than 8 y and younger than 20 y. |
| Setting and context | Varying settings, including school-based, community-based, and competitive sports (PA such as physical education and active transportation was excluded). |
| Description of intervention/phenomena of interest | An indicator of organized sports participation (involvement, frequency, volume, duration). |
| Search details |  |
| Number of databases sourced and searched | Five databases; Web of Science (Clarivate Analytics), SPORTDiscus (EBSCOhost), MEDLINE (PubMed), as well as ERIC and PsycINFO (ProQuest). Searches extended to the earliest date for each database. Search strategies were constructed uniquely for each database, yet all searches were focused on three groups of terms. Also, two supplemental steps included: (i)an adapted search through the Google Scholar search engine, with the first 500 results screened at the title and abstract level, and (ii)hand searches of reference lists of included studies and previous reviews of the literature. |
| Date range of database searching | A systematic search was conducted in October 2018 and was updated in April 2019. Searches extended to the earliest date for each database (e.g. Web of Science: 1900). |
| Publication date range of included studies | Most studies were published in the past 10 years, with the year of publication distribution in 5-year increments included 4% of studies published from 1996 to 2000, 7% from 2001 to 2005, 24% from 2006 to 2010, 24% from 2011 to 2015, and 41% of studies from 2016 to 2019. |
| Number and types of studies | 29 studies, of which 55% had a longitudinal design and 45% were cross-sectional. |
| Country of origin of included studies | Most studies originated from the US (n=10), Canada (n=9), Australia (n=3), and Spain (n=2). Several countries had one study included in the review (Iceland, Japan, Nigeria, and Slovenia) and one study included participants from various European countries. |
| Appraisal |  |
| Instruments used to appraise the primary studies and their quality | Risk of bias related to study methods, analyses, and reporting was assessed using an adapted 14-item coding tool. The coding tool was developed based on previous reviews (Eime et al., 2013; Evans et al., 2017), and integrated items for reporting, design, and measurement, as well as results and analyses. In addition to 12 items regarding study design and analysis from existing tools, the authors created two items regarding the sport participation operationalization that were unique to this review; these items assessed the clarity of the measurement approach. |
| Appraisal rating | All items were coded in a binary yes/no fashion and total scores for each article were calculated, with greater scores indicating a lower risk for bias. 83% of studies received a score between 11 and 14, indicating a  relatively low risk of bias. Noteworthy, items being least likely to be met, were the inclusion of a researcher-defined operationalization of sport involvement (72% unreported) and the reporting of actual p values (59% unreported). |
| Analysis | There was considerable variability in the way that sports participation was measured, hence four sub-groups were meta-analyzed separately (absence or presence of involvement, frequency of involvement, volume of involvement, and duration of participation), yet only when subgroups included enough effect sizes from distinct studies. |
| Methods of analyses | Effect sizes were quantitatively synthesized using random effects modelling, entailing that effect sizes were converted to correlation coefficients, prior computing an estimate of the mean population effect (i.e., ρ) that was weighted based on study sample size, and was corrected for unreliability in measurement (i.e., Cronbach’s α). Further, heterogeneity was considered, and when present, it was tested whether the correlation between sport participation and mental health was moderated by age or sex, and whether effect sizes differed significantly by the design-type of the study (i.e., primary data vs. secondary data use). Publication bias was examined by inspecting a contour-enhanced funnel plot of SEs with a quantitative estimate of bias (i.e., Egger’s regression test). |
| Outcome(s) assessed | A measure of anxiety and/or depression symptoms. |
| Results/findings | A total of 52 relevant effect sizes were included. |
| Significance/direction | Results from the random-effects meta-analyses indicated that symptoms of anxiety and depression were significantly lower among sport-involved adolescents than in those not involved in sport, although the effect size was small in magnitude. Significant effects were evident in the three analyses where enough studies had been published to permit meta-analysis: (i) anxiety associated with binary sport involvement, (ii) depression associated with binary sport involvement, and (iii) depression associated with either the frequency or volume of sport involvement. Effects were heterogenous and varied across study design and sample features, with moderation analyses revealing noteworthy trends related to age and sex. That is, inverse effects were stronger in samples that were relatively older, and studies including a greater proportion of male participants reported a stronger inverse correlation between sport involvement and anxiety symptoms. In addition, studies involving secondary analyses on existing data sets found a smaller inverse correlation between binary sport participation and depression symptoms, compared with studies in which the primary study aim was to examine the correlation between sport and depression symptoms.  Based on inspecting the contour-enhanced funnel plot of SEs, it was determined that the effect sizes did not deviate from symmetry enough to warrant concern for publication bias, and a nonsignificant Egger’s regression test indicated that publication bias was unlikely. |
| Heterogeneity | Large heterogeneity was identified, especially related to sport operationalizations, entailing sport involvement as a binary value, as well as frequency, volume, or duration of sport involvement, which in turn threatens generalizability of results. |
| *Comments:* | The authors conclude that although these results do not show a causal effect (due to survey-based studies, yet with prospective designs with large samples), they do support that sport participation during adolescence may represent a protective environment against anxiety and depressive symptoms. However, there is a need for increased knowledge on the psychosocial mechanisms that predict lowered anxiety and depression symptoms, as well as improved characterizations of sport contexts. |

| Study details | 8 |
| --- | --- |
| Title | **Influence of physical activity on bone strength in children and adolescents: A systematic review and narrative synthesis** |
| Author/year | **Tan et al., 2014** |
| Objectives | Primary aim was to determine the influence of PA and participation in organized sports on bone strength in children and adolescents. Secondary aims were to (i) identify sex-related or maturity-related differences regarding the influence of PA and participation in organized sport on bone strength, and (ii) assess the influence of PA and participation in organized sports on specific bone parameters (such as bone structure and density) that contribute to bone strength. |
| Type of review | Systematic review and narrative synthesis |
|  | In the following, data is extracted from only 13 out of the 37 included studies, i.e. observational studies of organized sports participation and bone strength, as 12/14 interventions were conducted at elementary schools, and 10 observational studies concerned recreational PA. Due to study heterogeneity, no meta-analysis was conducted (only a narrative synthesis), allowing for data extraction from 13 out of 37 studies. |
| Participant details (characteristics, total number) | Of the 13 studies that focused on organized sports, 11 assessed girls. Sample size of different sport groups ranged from n=9 to n=60 per group, and participants were aged five to 18 years. |
| Setting and context | Of the 13 studies that focused on organized sports, nine focused on gymnastics. Artistic and rhythmic gymnastics were included under the umbrella of gymnastics (6/9). Other studies assessed athletes who participated in tennis, running, soccer *and* swimming, or running *and* swimming *and* cycling *and* triathlon. |
| Description of intervention/phenomena of interest | Participation in organized sports |
| Search details |  |
| Number of databases sourced and searched | Seven databases; MEDLINE (from 1946), EMBASE (from 1974), CINAHL (from 1982), SportDiscus (from 1921), PEDro (from 1929), Informit (from 1977) and Cochrane Central Register of Controlled Trials. Text and and MeSH-terms were combined for the search concepts to create a detailed list of search terms. |
| Date range of database searching | ‘From’ varied across databases (see above), ‘to’ was January 17, 2013, for all. |
| Publication date range of included studies | 2002-2012 |
| Number and types of studies | 13 observational studies |
| Country of origin of included studies | Not explicitly reported |
| Appraisal |  |
| Instruments used to appraise the primary studies and their quality | The validated Quality Assessment Tool for Quantitative Studies, developed by the Effective Public Health Practice Project (EPHPP), was used to assess RCTs, CTs, and observational studies. |
| Appraisal rating | A weak, moderate, or strong global score for quality was allocated, based on six components: (1) selection bias; (2) study design; (3) confounders; (4) blinding; (5) data collection methods; and (6) withdrawals and dropouts. Based on these criteria, the global rating was strong if a study had four or more strong components (equal weights) and no weak components. If a study had less than four strong components and one weak component, the global rating was moderate, while if a study had two or more weak components, the global rating was weak. Of the 13 observational studies that focused on organized sports, 11 received a moderate global rating, while two were given a weak global rating. |
| Analysis |  |
| Methods of analyses | The diversity of measures used to assess bone strength and structure (eg, different imaging tools, measurement sites, and analysis protocols) across studies did not allow for a meta-analysis to be conducted. Thus, a narrative synthesis was conducted based on study design (intervention or observational studies). Within the observational studies (n=23), studies of organized sports (n=13) examined the influence of participation in a specific sport (training hours, type of training, age of training initiation) on bone outcomes. To avoid bias and encourage transparency, the methodological framework of Rodgers and colleagues was adopted, including elements of a narrative synthesis when a statistical meta‐analysis is not possible or advisable. |
| Outcome(s) assessed | Bone strength was the outcome of primary interest, whereas bone mass, structure, and microarchitecture were secondary outcomes. |
| Results/findings |  |
| Significance/direction | Athletes had significantly stronger bones (16% to 96%) compared with non-athletes across all 13 studies. This association was mediated by muscle. Bone structure was superior in athletes compared with non-athletes (values from 6% to 80% higher). Similarly, bone mass was 6% to 40% higher in athletes compared with non-athletes. Bone strength advantages for boys and girls engaged in sport were evident across all maturity groups. |
| Heterogeneity | There was substantial heterogeneity across studies in imaging tools used, anatomical sites measured, analysis procedures adopted, algorithms developed to estimate bone strength, as well as methods of assessing maturity, PA (questionnaire, accelerometry), and reported PA outcomes (PA type, frequency, etc.). Most studies evaluated prepubertal and peripubertal children. |
| *Comments:* |  |
