# Supplementary material for: Does organized sports participation in childhood and adolescence positively influence health? A review of reviews
Source: Prev Med Rep. 2021 May 30;23:101425. doi: 10.1016/j.pmedr.2021.101425 (PMC8190469; doi:10.1016/j.pmedr.2021.101425)
Supplement: Supplementary data 3 [file mmc3.docx]

**Excluded studies after full-text examination**

| Study | Reason for exclusion |
| --- | --- |
| Nelson et al., 2011 | Critical appraisal not conducted |
| Kwan et al., 2014 | Critical appraisal not conducted |
| Diehl et al., 2012 | Critical appraisal not conducted |
| Lisha and Sussman, 2010 | Critical appraisal not conducted |
| Jones et al., 2017 | Critical appraisal not conducted |
| Mays et al., 2011 | Critical appraisal not conducted |
| McIntosh-Dalmedo et al., 2018 | Violates the inclusion criteria on setting (extracurricular) and phenomenon of interest (organized PA, not PA per se) |
| Somerset and Hoare, 2018 | Barriers to participation in sports fall outside our main aim to assess associations with health |
| Venatsanou et al., 2015 | Critical appraisal not conducted |
| Allender et al., 2006 | Critical appraisal not conducted |
| Bean et al., 2014 | Critical appraisal not conducted |
| Mansfield et al., 2018 | Only 3/8 studies were conducted in youth <19 years, of which two were conducted in school settings |
| Gubbels et al. 2016 | Critical appraisal not conducted |
| Holt et al. 2017 | Critical appraisal not conducted |
| Gutierrez-Garcia et al. 2018 | Review concerning participation in judo exclusively |
